# Changes in vitamin D levels and depressive symptoms in later life in England

**DOI:** 10.1038/s41598-021-87432-3

**Published:** 2021-04-08

**Authors:** Giorgio Di Gessa, Jane P. Biddulph, Paola Zaninotto, Cesar de Oliveira

**Affiliations:** Department of Epidemiology and Public Health, University College London (UCL), 1-19 Torrington Place, London, WC1E 7HB UK

**Keywords:** Epidemiology, Epidemiology, Risk factors

## Abstract

Inadequate vitamin D levels have been associated with increased risk of depression. However, most of these studies are cross-sectional and failed to investigate the effect of changes in vitamin D levels. This study aimed to investigate the longitudinal association of changes in serum 25-hydroxyvitamin D levels with depressive symptoms in 3365 participants of the English Longitudinal Study of Ageing, a large nationally-representative study of older adults. Based on their vitamin D levels at baseline and follow-up (sufficient ≥ 50 nmol/L; insufficient < 50 nmol/L), participants were classified as follows: with sufficient levels at both waves; with sufficient levels at baseline but not at follow-up; with insufficient levels at baseline but ≥ 50 nmol/L at follow-up; and with levels < 50 nmol/L at each time point. Depressive symptoms were measured using the 8-point CES-D scale. Data were analysed using logistic regression models. Compared with those with sufficient levels of vitamin D at both waves, only those with insufficient levels throughout were more likely to report elevated depressive symptoms (OR = 1.39, 95% CI = 1.00–1.93). Becoming or no longer being vitamin D deficient was, in the short term, not associated with elevated depressive symptoms. Further evidence is required on whether vitamin D supplementation might contribute to the prevention or treatment of depression as well as on the duration of time for changes in vitamin D levels to lead to detectable changes in depressive symptoms.

## Introduction

It is estimated that depression affects more than 300 million people worldwide and that it is the world’s leading cause of disability^1^. Depression is also associated with higher mortality, reduced productivity, and increased direct and indirect healthcare costs^2–4^. Although depression aetiology and pathophysiology have not yet been fully explained, recent studies highlight the potential role of new biological factors that may affect mood. For instance, the presence of vitamin D receptors in the brain and its key role in the responses of the immune and nervous systems make it biologically plausible for vitamin D to be associated with brain activity and thus depression^5^, with hypovitaminosis D i.e. insufficiency or deficiency of vitamin D indicating an underlying biological susceptibility for depression^6^.

Hypovitaminosis D is a main public health problem worldwide. It is estimated that about 1 billion people worldwide have vitamin D deficiency, while 50% of the population has vitamin D insufficiency^7^ with a high prevalence of hypovitaminosis D also found in sunny climates^8,9^. Deficiency of 25-hydroxyvitamin D [25(OH)D], the major circulating form of vitamin D, is a widespread disorder across all age groups^10^. Older adults, however, are particularly at risk of hypovitaminosis D given that vitamin D absorption from food as well as skin synthesis by UVB-irradiation from sun exposure become less efficient with increasing age^9,11^.

Although vitamin D is well-known to be essential to maintain serum calcium and phosphorus levels in a healthy physiologic range for musculoskeletal health, observational studies have also found low vitamin D to be associated with a range of non-skeletal health conditions^9,12–14^ including symptoms of depression. Early work by Hoogendijk and colleagues^15^ using data from the Longitudinal Aging Study Amsterdam found that, among the 1282 adults aged 65–95 years, depressed subjects showed 25(OH)D levels that were 14% lower than non-depressed individuals (assessed using the Centre for Epidemiologic Studies Depression scale). With a few exceptions^16,17^, similar results have been found in other cross-sectional studies that have investigated the associations between depressive symptoms and vitamin D levels across different age groups, settings, and both among healthy non-institutionalised populations and in patients with medical comorbidities^18–24^.

However, cross-sectional studies do not allow determination of whether hypovitaminosis D precedes the onset of depression or whether individuals have low 25(OH)D levels because they are depressed. Cohort studies have tried to shed light on this issue exploring longitudinally the relationship between vitamin D and depression. A 2013 meta-analysis of three cohort studies reported mixed findings, depending on the categories and cut-off points being compared^25^. Other recent population-based longitudinal studies on both older men and women as well as adolescents also yielded inconclusive results^26–28^. Using data from the UK Biobank collected on participants aged between 40 and 69 years, Ronaldson and colleagues^29^ also found that vitamin D insufficiency at baseline was associated with depression at follow-up. All these longitudinal studies, however, have only examined the associations between vitamin D levels measured at one point in time (generally at baseline) and either changes in depression or depression at follow-up, failing to account for changes in vitamin D levels over time. This is an important oversight as seasonal changes in vitamin D levels are quite common^30^. Understanding whether changes in vitamin D levels are associated with changes in depressive symptoms helps to establish whether vitamin D plays a role in the prevention and/or treatment of depression. Thus, using data from the English Longitudinal Study of Ageing (ELSA), we aimed to examine whether and how changes in Vitamin D are associated with depressive symptoms among older men and women.

## Results

### Overall levels of Vitamin D and changes over time

Table 1 provides an overview of Vitamin D levels at baseline and follow-up, including changes over time and details by seasons. Overall, the average level of 25(OH)D was similar at both baseline and follow-up (at 50.2 nmol/L). As expected, both at baseline and follow-up, mean serum 25(OH)D concentration levels were insufficient when blood samples were collected in winter and spring compared to summer and autumn. When we looked at changes over time, 37% of ELSA respondents had insufficient levels of Serum 25(OH)D at both baseline and follow-up, with 31% presenting values > 50 nmol/L at both waves. The percentage of respondents with sufficient levels at baseline but not at follow-up (15%) was similar to that of respondents whose 25(OH)D increased over 50 nmol/L over time (16%). A seasonality effect can be seen with regards to both baseline values and changes: 46% of the sample whose blood was collected in the summer or autumn at baseline had insufficient levels of vitamin D at baseline compared to 69% of respondents whose initial interviews were in winter or spring.

**Table 1:** Changes in hypovitaminosis D and mean vitamin D level at baseline and follow-up—all sample and by baseline season.

	All seasons				Baseline season	
	% (n)	Mean vitamin D level at baseline nmol/L (SD)	Mean vitamin D level at follow-up nmol/L(SD)	Mean difference in vitamin D level nmol/L(SD)	Summer and Autumn % (n)	Winter and Spring % (n)
**Vitamin D transition category**						
25(OH)D > 50 nmol/L at baseline and follow-up	31.1 (1045)	73.1 (18.2)	71.6 (16.5)	− 1.5 (19.5)	35.1 (787)	23.2 (258)
25(OH)D ≤ 50 nmol/L at baseline and follow-up	37.4 (1255)	32.2 (10.6)	30.8 (10.9)	− 1.3 (11.9)	33.0 (739)	46.3 (516)
25(OH)D > 50 only at baseline	15.1 (506)	63.4 (12.5)	37.0 (9.6)	− 26.4 (15.4)	18.4 (413)	8.3 (93)
25(OH)D > 50 only at follow-up	16.4 (549)	35.6 (10.0)	66.1 (14.2)	+ 30.5 (18.0)	13.5 (301)	22.2 (248)
All	100 (3355)	50.20^1^ (23.0)	50.23^2^ (22.9)	0.03 (22.8)	100 (2240)	100 (1115)

^1^Mean vitamin D level in Summer and Autumn samples at baseline was 54.3 nmol/L; in Winter and Spring samples at baseline was 41.9 nmol/L.

^2^Mean vitamin D level in Summer and Autumn samples at follow-up was 54.9 nmol/L; in Winter and Spring samples at follow-up was 45.3 nmol/L.

Source: ELSA waves 6, 8, and 9. Analyses restricted to the sample with no missing values in any of the covariates used in the regression models.

### Baseline characteristics by follow-up depressive symptoms

Table 2 shows baseline characteristics by depressive symptoms at follow-up. Ten percent of the sample reported four or more depressive symptoms at follow-up, with almost half of respondents depressed at follow up having serum 25(OH)D levels below 50 nmol/L at both waves. Women, unpartnered respondents, those with lower levels of wealth, sedentary lifestyle, smokers, participants who reported more chronic diseases or ADL or IALD disabilities, those with high waist circumferences as well as participants with elevated depressive symptoms at baseline were more likely to report elevated depressive symptoms at follow-up (see Table 2 for full details).

**Table 2:** Baseline characteristics by depressive symptoms at follow-up.

	CES-D score < 4	≥ 4 CES-D symptoms	*P* value
**Change in vitamin D levels (%)**			
25(OH)D > 50 nmol/L at both waves	32.1	21.9	< 0.001
25(OH)D ≤ 50 nmol/L at both waves	36.2	48.7	
25(OH)D > 50 only at baseline	15.5	11.7	
25(OH)D > 50 only at follow-up	16.2	17.7	
**Mean age (SD)**	65.4 (8.31)	65.6 (8.96)	0.661
**% Men**	46.1	29.4	< 0.001
**Wave 8 follow-up interview**	66.7	68.5	0.495
**Partnered**	70.7	53.5	< 0.001
**Education**			
Low	33.4	43.2	< 0.001
Medium	46.3	44.5	
High	20.3	12.3	
**Wealth quintiles**			
Lowest	12.5	27.0	< 0.001
2nd	14.8	24.0	
3rd	21.1	21.0	
4th	24.7	16.9	
Highest	26.9	11.1	
**Physical activity**			
Sedentary or low	7.5	22.5	< 0.001
Moderate	42.7	50.5	
Vigorous	49.8	27.0	
**Smoking status**			
Never smoked	40.1	33.3	< 0.001
Former smoker	51.1	49.3	
Current smoker	8.7	17.4	
**Mean number of comorbidities (SD)**	1.02 (1.04)	1.61 (1.29)	< 0.001
**Mean number of ADLs or IADLs (SD)**	0.31 (1.00)	1.26 (1.99)	< 0.001
**Waist**			
Low	23.7	18.0	< 0.001
Medium	26.5	20.1	
High	49.8	61.9	
**≥ 4 CES-D symptoms at baseline**	6.5	41.4	< 0.001
**Mean number of baseline depressive symptoms (SD)**	0.89 (1.43)	3.27 (2.56)	< 0.001
**Mean number of depressive symptoms at follow-up (SD)**	0.75 (0.94)	5.26 (1.31)	< 0.001
**History of doctor-diagnosed depression**	5.7	19.5	< 0.001
**Summer or Autumn**	33.5	30.6	0.284
**Total % (and N) of respondents**	90.1 (3022)	9.9 (333)	

*Source*: ELSA waves 6, 8, and 9. Analyses restricted to the sample with no missing values in any of the covariates used in the regression models.

### Multivariate analyses

Odds ratios for the associations between changes in vitamin D levels and elevated depressive symptoms at follow-up are presented in Table 3. In the unadjusted analyses (Model 1) low levels of 25(OH)D at both baseline and follow-up were significantly associated with elevated depressive symptoms (OR = 1.97, 95% CI = 1.48–2.64). Moreover, those whose levels of vitamin D were insufficient only at baseline had higher odds of reporting elevated depressive symptoms at follow-up (OR = 1.60 95% CI = 1.12–2.30) compared with those with 25(OH)D levels above 50 nmol/L at both waves. After controlling for socio-demographic, economic, and health characteristics, only participants with hypovitaminosis D at both waves remained significantly more likely to have elevated depressive symptoms at follow-up than those with sufficient levels even, although the OR attenuated from Models 2 (OR = 1.96) to Model 5 (OR = 1.49). Even after controlling for elevated depressive symptoms at baseline and seasonality of interviews (Model 7), those with insufficient levels of vitamin D were more likely to report elevated depressive symptoms at follow-up compared with those with 25(OH)D levels above 50 nmol/L at both waves (OR = 1.39, 95% CI = 1.00–1.93). Across all models, those who became at risk (i.e. whose 25(OH)D levels dropped below 50 nmol/L) were not associated with elevated depressive symptoms at follow-up.

**Table 3:** Unadjusted and fully adjusted ORs (with 95% CIs) for logistic regression analyses investigating the association between changes in vitamin D levels and elevated depressive symptoms at follow-up (N = 3355).

	Model 1 (unadjusted)	Model 2	Model 3	Model 4	Model 5	Model 6	Model 7
	OR (95% CI)	OR (95% CI)	OR (95% CI)	OR (95% CI)	OR (95% CI)	OR (95% CI)	OR (95% CI)
**Changes in 25(OH)D**							
> 50 at both	Reference	Reference	Reference	Reference	Reference	Reference	Reference
≤ 50 at both	1.97 (1.48–2.64)	1.96 (1.47–2.62)	1.63 (1.21–2.20)	1.48 (1.09–2.02)	1.49 (1.09–2.04)	1.36 (0.98–1.88)	1.39 (1.00–1.93)
Became at risk	1.11 (0.74–1.67)	1.11 (0.74–1.67)	1.01 (0.67–1.53)	1.03 (0.68–1.58)	1.04 (0.68–1.58)	1.05 (0.68–1.62)	1.05 (0.68–1.62)
No longer at risk	1.60 (1.12–2.30)	1.56 (1.09–2.24)	1.35 (0.93–1.96)	1.30 (0.89–1.90)	1.30 (0.89–1.91)	1.17 (0.79–1.74)	1.17 (0.79–1.74)

OR = Odds Ratio; CI = Confidence Interval. Nested models, with Model 1 unadjusted; Model 2 = Model 1 + adjusted for age, sex and the wave at follow-up; Model 3 = Model 2 + adjusted for partnership, paid work, education, and wealth. Model 4 = Model 3 + adjusted for health behaviours, health conditions, and functional limitations; Model 5 = Model 4 + adjusted for waist circumference; Model 6 = Model 5 + baseline elevated depressive symptoms and history of doctor-diagnosed depression; Model 7 = Model 6 + season of blood collection at follow-up and an indicator of same seasonality across waves. Source: ELSA waves 6, 8, and 9.

Sensitivity analyses (Table 4) were used to further check the robustness of our results. Results showed that even taking into account changes in partnership, employment, wealth, and health (Model A), we found that respondents with low vitamin D levels across waves had significantly higher odds of elevated depressive symptoms at follow-up (OR = 1.46, 95% CI = 1.05–2.03). Similarly, when we restricted the sample to participants who were interviewed in the same season at both baseline and follow-up (Model B), those with hypovitaminosis D across waves were significantly more likely to report elevated depressive symptoms (OR = 1.52, 95% CI 1.01–2.29) than those with 25(OH)D levels above 50 nmol/L at both interviews. When using the number of timepoints (0, 1, or 2) ‘at risk’ through insufficient levels of Vitamin D, we found that having 25(OH)D levels ≤ 50 nmol/L only once (that is either at baseline or follow-up) was not associated with elevated depressive symptoms at follow-up. Furthermore, when baseline levels of vitamin D and absolute changes were considered, we found that absolute changes in 25(OH)D were not associated with elevated depressive symptoms at follow-up. Results suggesting that hypovitaminosis D across waves was associated with elevated depressive symptoms were confirmed even when analyses were restricted to respondents with no elevated depressive symptoms and no history of doctor-diagnosed depression at baseline (Model C).

**Table 4:** Unadjusted and fully adjusted ORs (with 95% CIs) for the relationship between three different indicators of changes in vitamin D levels and elevated depressive symptoms at follow-up under different model assumptions.

	Model A		Model B		Model C	
	Unadjusted	Fully adjusted	Unadjusted	Fully adjusted	Unadjusted	Fully adjusted
	OR (95% CI)	OR (95% CI)	OR (95% CI)	OR (95% CI)	OR (95% CI)	OR (95% CI)
**Changes in 25(OH)D**						
> 50 nmol/L at both	Reference	Reference	Reference	Reference	Reference	Reference
≤ 50 nmol/L at both	1.97 (1.48–2.64)	1.46 (1.05–2.03)	2.20 (1.54–3.14)	1.52 (1.01–2.29)	2.21 (1.48–3.27)	1.93 (1.27–2.94)
Became at risk	1.11 (0.74–1.67)	1.05 (0.68–1.63)	1.40 (0.85–2.32)	1.29 (0.75–2.23)	1.19 (0.69–2.06)	1.14 (0.64–2.00)
No longer at risk	1.60 (1.12–2.30)	1.27 (0.84–1.89)	1.67 (1.07–2.62)	1.27 (0.77–2.11)	1.70 (1.03–2.78)	1.56 (0.93–2.59)
**N of times at risk of hypovitaminosis D**						
Never	Reference	Reference	Reference	Reference	Reference	Reference
Once	1.36 (0.99–1.87)	1.17 (0.82–1.66)	1.55 (1.05–2.30)	1.27 (0.83–1.95)	1.44 (0.94–2.23)	1.35 (0.86–2.11)
Twice	1.97 (1.48–2.64)	1.46 (1.05–2.03)	2.20 (1.54–3.14)	1.52 (1.01–2.29)	2.21 (1.48–3.27)	1.93 (1.27–2.94)
**Baseline 25(OH)D and absolute change**						
> 50 nmol/L	Reference	Reference	Reference	Reference	Reference	Reference
≤ 50 nmol/L	1.77 (1.39–2.34)	1.35 (1.01–1.81)	1.77 (1.36–2.29)	1.37 (0.95–1.97)	2.01 (1.41–2.85)	1.79 (1.23–2.59)
Difference in 25(OH)D over time	1.00 (0.99–1.01)	1.00 (0.99–1.01)	1.00 (0.99–1.01)	1.00 (0.99–1.01)	1.00 (0.99–1.01)	1.00 (0.99–1.01)
N respondents	3324		2251		2863	

*OR *odds ratio, *CI* confidence interval. Model A refers to analyses which adjust for changes in respondents’ partnership, employment, wealth, and health instead of considering these variables only at baseline. The fully-adjusted Model A also adjusts for age, sex, wave at follow-up, education, waist circumference, baseline elevated depressive symptoms, history of doctor-diagnosed depression, season of blood collection at follow-up, and an indicator of same seasonality across waves. Model B only considers those whose blood samples were collected in the same season (winter or spring vs summer or autumn) at both baseline and follow-up. The fully adjusted Model B adjusts for age, sex, wave at follow-up, partnership, paid work, education, wealth, health behaviours, health conditions, functional limitations, waist circumference, baseline elevated depressive symptoms, history of doctor-diagnosed depression and season of blood collection at follow-up. Model C only considers those with no elevated depressive symptoms and no history of doctor-diagnosed depression at baseline. The fully adjusted Model C adjusts for age, sex, wave at follow-up, partnership, paid work, education, wealth, health behaviours, health conditions, functional limitations, waist circumference, season of blood collection at follow-up and an indicator of same seasonality across waves. Source: ELSA waves 6, 8, and 9.

## Discussion

Cross-sectional associations between vitamin D insufficiency and depression or depressive symptoms are well documented but few studies have investigated this relationship longitudinally. To our knowledge, no prior studies have looked at the associations between changes in vitamin D levels and depressive symptoms. Using ELSA’s longitudinal and large representative sample of older adults aged 50 years and older, we found that compared with participants with sustained sufficient levels of vitamin D, those with persistent hypovitaminosis D over time have increased odds of reporting elevated depressive symptoms, even when a range of socio-economic and health characteristics, including baseline elevated depressive symptoms, are accounted for. However, our study does not provide evidence that ‘becoming at risk’ of hypovitaminosis D or ‘being no longer at risk’ is associated with changes in depressive symptoms.

Our results are in line with other observational longitudinal studies which found that baseline low vitamin D was associated with subsequent depression among older people^25,28,29^. Several direct and indirect biological mechanisms have been hypothesised to explain these associations, including the presence of Vitamin D receptors in areas of the brain associated to the development of depression, its role in the formation of serotonin, and its proposed anti-inflammatory effect^31,32^.

Our results also suggest that Vitamin D status is not stable over time for a considerable percentage of older people; however, in our study we found that changes in vitamin D levels were not associated with changes in elevated depressive symptoms. This finding warrants further studies on the effect of changes in Vitamin D levels on depression, and more specifically about the duration of such changes. In our study, where vitamin D level was assessed on two single time points, we cannot establish when and for how long respondents have become/are no longer at risk of hypovitaminosis D. Both factors, however, are likely to play an important role in understanding whether and how long it takes for changes in Vitamin D to affect depressive symptoms, as suggested by studies that have investigated the role of Vitamin D supplementation as prevention or treatment for later life depression. A randomised controlled trial conducted by Sanders and colleagues^33^, which investigated the effects of a yearly, large dose of 500,000 IU of vitamin D3 on depressive symptoms in women aged 70 years and older, concluded that although serum 25(OH)D levels were 41% higher in the women who received the vitamin D supplements versus the placebo after one year, no significant change in mood or mental well-being was found. Jorde and colleagues^34^, however, found in their trial of overweight and obese men and women aged 21–70 years that there was evidence of modest improvement in depression scores after one year of vitamin D supplementation. More recent studies and systematic reviews^35–39^ continue to provide mixed findings to support the role of vitamin D supplementation in depression, depending on the target population, the duration, the definition of depression, as well as the dosage used.

Nonetheless, given seasonal changes in the levels of vitamin D, supplementation may be important throughout the year, and in the winter in particular. Given the association of hypovitaminosis D with a number of indicators of poor health and musculoskeletal disorders, particularly among older adults, it is important to maintain sufficient levels of vitamin D over time, and this may be challenging when sun exposure is limited or inadequate. Small randomised trials also suggest that supplementation during winter with vitamin D might help seasonal mood disorders and improve well-being^40–42^.

The strengths of the study include the use of longitudinal data from a large and representative sample of community-dwelling men and women aged 50 years and older in England. We were also able to adjust our analyses for a wide range of socio-economic and health variables. Moreover, blood collection and analyses followed standardised protocols that ensure data quality. Our study, however, has some limitations and whilst this study is the first to assess changes in Vitamin D levels, we only had individual data pertaining to two timepoints. Therefore, we cannot ascertain the duration of hypovitaminosis D; this could be particularly important for those who ‘became at risk’ or ‘were no longer at risk’ of low vitamin D levels. As ELSA plans to continue to collect blood samples, future analyses will allow for a more nuanced and greater understanding of the dynamics of changes in Vitamin D and depressive symptoms. Moreover, the study may suffer from potential selection bias through eligibility criteria for the nurse visit, which was limited to those consenting to have their blood taken, and those who have remained in the study. However, if healthier respondents were more likely to both give blood and stay in the study, it is possible that our estimates may be conservative. Finally, ELSA does not collect information on the general use of vitamin D supplementation.

In conclusion, our study suggests a role of vitamin D in depression, with those with consistent hypovitaminosis D more likely to be depressed than those with high levels of vitamin D. Given the high prevalence of hypovitaminosis D and depression in later life, our findings might have significant public health implications. However, additional data from randomised controlled trials of Vitamin D supplementation are needed to ascertain if this could be a cost-effective strategy to prevent and reduce depression in later life.

## Methods

### Study population

We used data from the English Longitudinal Study of Ageing (ELSA), a multidisciplinary longitudinal survey representative of men and women aged 50 and over living in private households in England^43^. The first interview took place in 2002/03, with biennial follow-up interviews and objective health examinations for all members (i.e. nurse visits) every 4 years until wave 6. Across wave 8 and 9, however, a new approach was taken when designing the nurse visit and two mutually exclusive subsets of members were selected: one was offered a nurse visit at wave 8, and the other in wave 9. Full details of the study, its sampling design, and technical reports can be found elsewhere (http://www.elsa-project.ac.uk/). Analyses for this study used data from waves 6, 8, and 9 collected in 2012/13, 2016/17, and 2018/19, respectively. Wave 6 of ELSA was the first to measure Vitamin D. In our analytical sample, we included participants who had a valid assessment of 25OHD at both baseline (wave 6) and follow-up (either wave 8 or 9) and measures of depression and the other covariates at both time points (please, see sections below). The analytical sample for the longitudinal analysis consisted of 3365 individuals. All participants gave written informed consent. The National Research Ethics Service (London Multicentre Research Ethics Committee [MREC/01/2/91]) has approved the ELSA study.

### Assessment of 25OHD

At Wave 6, of the 7730 who had a nurse visit, blood samples were obtained from 6206 participants. Blood samples were not taken from respondents who had a clotting or bleeding disorder (e.g., haemophilia or low platelets), had ever had a fit, were on anticoagulant drugs (e.g., warfarin therapy) or did not give their consent in writing. 25(OH)D concentrations were ascertained in 5870. Almost 60% of these respondents (N = 3472) also had a valid assessment of the vitamin D level at follow-up (either at Wave 8 or Wave 9). All blood samples were analysed at the Victoria Infirmary (Newcastle-upon-Tyne, United Kingdom). The vitamin D level was assessed by measuring the plasma level of 25(OH)D. This has a circulating half-life of 2–3 weeks and is not influenced by changes in calcium and parathyroid hormone levels and is a reliable indicator of vitamin D status^44^. Serum 25(OH)D levels were measured by the Diasorin Liaison immunoassay that detects both 25(OH)D2 and 25(OH)D3 and therefore, provides the total circulating 25(OH)D level. The assay for 25(OH)D has an analytical sensitivity (lower detection limit) of 7.5 nmol/L. The detection limit represents the lowest measurable analyte level that can be distinguished from zero. All assays were performed in duplicate. The coefficient of variation ranged from 8.7 to 9.4%. The laboratory performing the 25(OH)D analyses took part in the Internal and the Vitamin D External Quality Assessment Schemes (DEQAS). Based on the recommendations made by advisory bodies such as the European Food Safety Authority^45^ or the Institute of Medicine^46^ we categorised 25(OH)D levels using the truncated categorisation of ≤ 50 nmol/L (insufficient levels) and > 50 nmol/L. In order to examine changes in Vitamin D levels between waves of the survey we distinguished between those with sufficient levels at both waves; those who became at risk (with sufficient levels at baseline but not at follow-up); those no longer at risk (with insufficient levels at baseline but with 25(OH)D levels > 50 nmol/L at follow-up) and those with insufficient levels of Vitamin D at both time points.

### Assessment of depressive symptoms

Depressive symptoms were measured by a short eight-item version of the validated Centre for Epidemiologic Studies Depression (CES-D) Scale. The CES-D scale is not a diagnostic instrument for clinical depression but can be used to identify people “at risk” of depression in population-based studies^47^. This short version had good internal consistency at each wave (Cronbach’s α > 0.95) and comparable psychometric properties to the full 20-item CES-D^48^. Respondents were asked whether they had experienced any depressive symptoms, such as restless sleep or being unhappy in the week prior to interview. Respondents who reported four or more depressive symptoms in the week prior to the interview were classified as reporting elevated depressive symptoms^49,50^.

### Covariates

A wide range of potential health, demographic, and socioeconomic confounders known to be associated with either depression or the likelihood of having low 25(OH)D levels were included. We controlled for baseline respondent’s age, gender, partnership status, employment status, education, and quintiles of total net non-housing wealth. Age squared was not included in the model as adding this as a predictor variable did not result in a statistically significant improvement in model fit (p value for the likelihood ratio test was 0.341). Health behaviour measures included smoking status (non-smoker, former smoker, or current smoker) and self-reported physical activity (sedentary or low, moderate, or vigorous). Health measures included a summary score adding up the number of self-reported doctor diagnosed chronic conditions (high blood pressure, angina, heart attack, heart failure, heart murmur, hearth rhythm, diabetes, cancer, stroke, arthritis, lung diseases, or Parkinson’s) as well as the sum of any difficulties with 13 basic (ADL) and instrumental (IADL) activities of daily living. A history of doctor diagnosed depression was established by asking participants whether they were ever told by a doctor they had depression. Waist circumference was categorised into three main groups using sex-specific thresholds: low (< 94 cm for men; < 80 cm for women), medium (≥ 94 cm and < 102 cm for men; ≥ 80 cm and < 88 for women), and high (≥ 102 cm for men; ≥ 88 cm for women). We also included the season when the follow-up sample was collected, and an indicator capturing whether follow-up blood sample was collected in the same grouped season (summer and autumn or winter and spring) or not. Finally, we controlled for an indicator of the wave of the follow-up interview.

### Statistical analysis

Following descriptive findings to explore the baseline characteristics of the study population, longitudinal logistic regression models were used to determine whether changes in vitamin D levels were associated with depression. Following the unadjusted model (Model 1), a further six models adjusted for potential confounders were performed, with each subsequent model including variables from the previous model. Model 2 adjusted for age, sex, and the wave at follow-up. Model 3 further adjusted for partnership, employment status, education, and wealth; Model 4, for health behaviours, health conditions, and functional limitations; Model 5, for waist circumference; Model 6, for elevated baseline depressive symptoms and history of doctor-diagnosed depression; and finally, Model 7, for the season of blood collection on follow-up, and the seasonal indicator variable. All analyses were carried out using Stata SE version 15.1.

### Sensitivity analyses

Sensitivity analyses were conducted to test the robustness of our models. In particular, we repeated our analyses (a) taking into account changes in respondents’ partnership, wealth, and health instead of considering these variables only at baseline; (b) restricting the sample to those who had had their blood sample taken in the same seasons at both baseline and follow-up; and (c) restricting the sample to those without a history of doctor-diagnosed depression or elevated depressive symptoms at baseline to rule out potential reverse causation of the associations. We also repeated analyses by using as the main variable of interest the number of times ‘at risk’ of having insufficient levels of Vitamin D (distinguishing between never, once, and at both waves) as well as considering baseline hypovitaminosis D and absolute changes in Vitamin D levels over time.

## Data Availability

The data were made available through the UK Data Archive.
